# Treatment Effects of Jinlingzi Powder and Its Extractive Components on Gastric Ulcer Induced by Acetic Acid in Rats

**DOI:** 10.1155/2019/7365841

**Published:** 2019-01-03

**Authors:** Xueying Zhao, Ji Li, Yonghai Meng, Mingming Cao, Jianwei Wang

**Affiliations:** School of Basic Medical Sciences, Heilongjiang University of Chinese Medicine, 24 Heping Road, Harbin, 150040, China

## Abstract

Jinlingzi powder comprises* Melia toosendan Sieb. et Zucc*. and* Corydalis yanhusuo (Y.H. Chou & Chun C.Hsu) W.T. Wang ex Z.Y. Su & C.Y. Wu* and is usually applied in clinic as traditional Chinese medicine for pain. The present study aims to investigate the therapeutic actions of Jinlingzi powder and its extracted components and theirs treatment mechanism on the acetic acid induced-gastric ulcer in rats. The gastric ulcer model was induced by the administration of acetic acid in rats (84 male). Jinlingzi powder water decoction, its polysaccharide, and nonalkaloid and alkaloid components were used to investigate the therapeutic actions on the acetic acid induced-gastric ulcer by measuring the related pharmacy and pharmacodynamic factors, including ulcer index, ulcer area, ulcer healing rate, interleukin-8 (IL-8), tumor necrosis factor-*α* (TNF-*α*), neurotensin (NT), platelet activating factor (PAF), thromboxane B2 (TXB2), and vascular endothelial growth factor (VEGF) in rat serum, acetylcholinesterase (AChE) in brain tissue, prostaglandin E2 (PGE2), and basic fibroblast growth factor (bFGF) in gastric tissue. Among the various groups, Jinlingzi powder and the nonalkaloid components caused significant changes in IL-8, TNF-*α*, NT, PAF TXB2, and VEGF values in the serum. The AChE content in the rats' brain tissue was also reduced after using Jinlingzi powder and the nonalkaloid components. Additionally, Jinlingzi powder and the nonalkaloid components considerably affect the amount of PGE2 and bFGF in a rat's stomach tissue. Therefore, Jinlingzi powder and the nonalkaloid components can effectively inhibit neutral neutrophil activation, prevent capillaries thrombosis, and protect gastric mucosa. Thus, the nonalkaloid components of the Jinlingzi powder play a key role in the treatment of gastric ulcer.

## 1. Introduction

Gastric ulcer, a type of local tissue defect caused by various factors, is a common disease worldwide [1]. It is usually derived from the mucosa, submucous, muscle, and serosa layers of stomach, which are soaked in the gastric acid, gastric juice, and pepsinum. At least 10% of the world's population are troubled by this disease in their lifetime [2, 3].

The healing of gastric ulcer is a complex process that requires not only the filling of the mucosa but also tissue reconstruction under the mucosa [2] and is closely related to the gastrointestinal hormone and cytokine that can regulate the gastrointestinal motility affecting the secretion of gastric acid and pepsin, which play a mucosal protective role. In the past few years, substantial researches have been conducted on gastrin, somatostatin, epidermal growth factor, neurotensin, and the faded leather hormone [4, 5]. Gastrin can directly act on the gastric parietal cell to promote the secretion of gastric acid, whereas somatostatin can inhibit the secretion of gastrin, stomach acid, and pepsin. The epidermal growth factor can promote the growth of gastric mucosal cells [4]. Meanwhile, neurotensin and cerulein can protect the cells. Some cell growth factors that are related to the secretion of gastric acid, cell migration, cell differentiation, cell proliferation, and formation of extracellular matrix and new vessels can participate in the healing process of gastric ulcer [4]. Basic fibroblast growth factor (bFGF) and vascular endothelial growth factor (VEGF) play a crucial role in tissue remodeling, and formation of extracellular matrix and new vessels [4, 6–10].

Medicines for gastric ulcer treatment are mainly used for relieving symptoms, promoting ulcer healing, preventing ulcer relapse, and avoiding complications. So far, two kinds of medicines are being used in clinical practice: those for treating hyperacidity and for protecting the gastric mucosa [11–13].

In Chinese medicine, Jinlingzi powder has been widely used in clinical departments 1000 years ago for the alleviation of pain, such as epigastric, ribs, and abdominal pain. Many researchers have applied Jinlingzi powder to treat gastropathy such as gastric ulcer, chronic superficial gastritis, chronic atrophic gastritis, bile reflux gastritis, alkaline reflux gastritis, chronic erosive gastritis, and chronic gastritis infected by campylobacter pylori. Good treatment results have been produced by using Jinlingzi powder for the above gastropathy. In 2002, Ye et. al. reported a detailed summary of the clinical application of Jinlingzi powder [14]. Ding and Zhang studied 104 clinical cases in which Jinlingzi powder showed a good curative effect on stomachache [15]. Wang et. al. summarized the research progress of Jinlingzi powder including dosage forms, quality standards, compatibility and efficacy, and clinical application progress [16]. The correlation between the traditional and modern research on the function of Jinlingzi powder was discussed based on the literature and clinical results [17]. Recently, Shen et. al. reviewed studies on Jinlingzi powder in traditional Chinese medicine [18]. We have also obtained good treatment effects in the clinical application of Jinlingzi powder for the treatment of the epigastric pain [19]. In addition, Jinlingzi powder also exhibited a remarkable effect on the pain-relieving model in mice which was established by using the hot plate and acetic acid writhing methods in our study [20].

Generally, Jinlingzi powder comprises* Melia toosendan Sieb. et Zucc.* and* Corydalis yanhusuo (Y.H. Chou & Chun C.Hsu) W.T. Wang ex Z.Y. Su & C.Y. Wu* with a mass ratio of 1:1.* Melia toosendan Sieb. et Zucc.* mostly contains toosendanin and other terpenoids, alkaloids, flavone, and tannin, whereas* Corydalis yanhusuo (Y.H. Chou & Chun C.Hsu) W.T. Wang ex Z.Y. Su & C.Y. Wu* is rich in berberine type alkaloids [18]. Although Jinlingzi powder has a good clinical effect on treating stomachache, the material basis for its efficacy and mechanism of action are still unclear due to its complex element.

In this study, Jinlingzi powder is applied for the treatment of gastric ulcer induced by acetic acid. Furthermore, Jinlingzi powder is preliminarily divided into polysaccharide (C1), nonalkaloid (C2), and alkaloid (C3) components by the extraction with ethanol and water. In addition, the therapeutic actions of Jinlingzi powder and its components on the experimental model of gastric ulcer induced by acetic acid are investigated by measuring related pharmacy and pharmacodynamic factors. Thus, the treatment mechanism of the Jinlingzi powder decoction and its effective components is proposed.

## 2. Materials and Methods

### 2.1. Jinlingzi Powder Material

The dried ripe fruit of* Melia toosendan Sieb. et Zucc*. and the dried tuber of* Corydalis yanhusuo (Y.H. Chou & Chun C.Hsu) W.T. Wang ex Z.Y. Su & C.Y. Wu* were purchased from the Second Affiliated Hospital of Heilongjiang University of Chinese Medicine. The specimen was identified and authenticated by Jianwei Wang, a professor of Heilongjiang University of Chinese Medicine, and the voucher specimens of the plants were deposited at the School of Basic Medical Sciences of the university.

### 2.2. Water Decoction of Jinlingzi Powder

250 g of* Melia toosendan Sieb. et Zucc*. and 250 g of* Corydalis yanhusuo (Y.H. Chou & Chun C.Hsu) W.T. Wang ex Z.Y. Su & C.Y. Wu* were boiled with 2,000 g water using soft fire twice, for 1.5 h each. Then the filtrate was condensed into 60.4 g of dry paste. A soup of Jinlingzi powder with a concentration of 0.13 g/mL was obtained by using deionized water to dilute the dry paste. The soup was placed in a 250 mL glass bottle and was kept at low temperature.

### 2.3. Extraction and Identification of the Effective Components of Jinlingzi Powder

The effective components of Jinlingzi powder were extracted using ethanol and water. Briefly, 2.5 Kg of* Melia toosendan Sieb. et Zucc*. and 2.5 Kg of* Corydalis yanhusuo (Y.H. Chou & Chun C.Hsu) W.T. Wang ex Z.Y. Su & C.Y. Wu* were added to 8 times the amount of water, soaked for 30 min, and then boiled with soft fire for 2 h. The mixture was filtrated to collect the filtrate and filter residue. 6-time amount of water in the precipitate was added, and the mixture was boiled with soft fire for 2 h again. After filtration, the above filters were put together and condensed into 5,000 mL of liquid. 95% ethanol was added to the concentrated liquid until the concentration of ethanol became 75%. Then, the mixture was settled for 24 h and was filtered. The precipitate was washed and dried with 75% ethanol to obtain crude polysaccharide components (C1); the supernatant underwent reduced pressure distillation to remove ethanol, and then the volume of the above liquid was increased to 5,000 mL by adding water. Finally, hydrochloric acid was added to the solution, and the concentration of hydrochloric acid was maintained to 5%. The above solution passed through a cation exchange resin column. The pH value of the obtained ion exchange liquid was adjusted to 7 by adding NaOH. The nonalkaloid components (C2) were obtained by concentrating the liquid. The alkali absorption resin was dried and alkalified with ammonia, and then it was refluxed and extracted with 95% ethanol again and again. After merging the extracting solution, the ethanol was removed from the solution via reduced pressure distillation to obtain alkaloid components (C3).

To assure the repeatability of this pharmacological study, the extract experiment was repeated more than three times and the obtained components C1, C2, and C3 were identified via chemical method and thin-layer chromatography.

### 2.4. Animals

84 clean grade SD rats (male), which were provided by the Experimental Animal Center of Heilongjiang University of Chinese Medicine, were used in the experiments. The weight of each rat was in the range of 180-220 g. The rats were housed in a laboratory under standard conditions as follows: standard food and water, temperature at 18-22°C, humidity at 50-60%, airflow rate of 10-25 cm/s, and noise below 50 decibels. Before the experiment, the rats were fed for 7 days in laboratory. All animal experimental procedures were performed in accordance with the Regulations for the Administration of Affairs Concerning Experimental Animals approved by the State Council of People's Republic of China. All animal studies were approved by the Ethics Committee for Animal Research of Heilongjiang University of Chinese Medicine (Protocol number: 2016061901). The Minimum Standards of Reporting Checklist (Supplementary Materials section) contains details of the experimental design, statistics, and resources used in this study.

### 2.5. Animal Grouping and Modeling

12 rats were randomly selected and denoted as blank group (BG). The other rats were induced with acetic acid to make a gastric ulcer model. Chronic gastric ulcers were induced in rats according to the method in the literature [21, 22] with slight modifications. Briefly, the rats cannot be fed except with water for 24 h before they were made as model. First, the rats were given a general anesthetic with diethyl ether. Second, they were placed on rat holders in a supine position for the cutting of the side hair and sterilization. Third, the abdominal wall was cut about 3 cm hierarchically under the sternum following the abdominal line slightly to the left, and the stomach was taken out to expose its glands. Fourth, the rats' stomachs were attached to a plastic pipe with diameter of 6 mm and length of 2 cm and were fixed using a self-made stomach clamp. Next, 0.10 mL of 100% glacial acetic acid was injected into the chorion of the anterior wall of the stomach to ensure the contact time for 60 s before removing the stomach clamp. Then, the residual glacial acetic acid was wiped slightly with dry cotton swabs and physiological saline. Finally, after covering omentum in some regions, the stomach was carefully placed back in the enterocoelia, and the incision was sutured and sterilized with physiological saline and ethanol. According to different medical treatments, these molding rats were randomized into six groups, with 12 rats per group: model group (MG), omeprazole group (OG), Jinlingzi powder group extracted using water (JG), crude polysaccharide group (C1G), nonalkaloid group (C2G), and alkaloid group (C3G).

### 2.6. Dosages of Different Groups

The BG and MG were given 0.9% physiologic saline; the OG was supplied with 0.36 mg/mL omeprazole enteric-coated-capsules that were purchased from Renmintongtai Pharmaceutical Co., Ltd., (Harbin, China) (Chinese drug approval number: J20130092); the JG was fed with 0.13 g/mL of Jinlingzi powder soup; the C1G, C2G, and C3G were given 0.16, 0.234, and 4.5 mg/mL of soups of crude polysaccharide, nonalkaloid, and alkaloid components, respectively. Each group was fed a dose of medicine once a day for 12 days, and the dose volume was 1 mL/100 g according to the weight of the rats.

### 2.7. Detection Index and Methods

Ulcer index (UI), ulcer area (UA), and ulcer healing rate (UHR) were measured using the following method: After taking the rat's blood, the rats were killed by taking off their neck. The gastric tissue was cut from the cardia to the pylorus after carefully cutting the abdomen to expose gastric tissue. Then the gastric tissue was scissored along the greater curvature and washed clean with physiological saline. Finally, the scissored gastric tissue was placed on glass pane to measure the largest vertical and transverse diameter crossing ulcer center with Vernier caliper under dissecting microscope.

The UI was the sum of the largest vertical diameter (d1) and the largest transverse diameter (d2) crossing ulcer center.

The UA was calculated as

(1) $$\begin{array}{c} UA=\pi \times \left(\;\frac{dl}{2}\;\right)\times \left(\;\frac{d2}{2}\;\right) \end{array}$$

and the UHR was calculated as

(2) $$\begin{array}{c} UHR\;\left(\;\%\;\right)=\frac{\left(UI_{MG}-UI_{EG}\right)\times 100\%}{UI_{MG}} \end{array}$$

where UI_MG_ and UI_EG_ represent the sum of UI mean value of MG and the sum of UI mean value of the experimental group, respectively.

IL-8, TNF-*α*, NT, PAF, TXB2, VEGF, AChE, PGE2, and bFGF were measured by using corresponding kits (R&D Systems, USA). According to the operation method of the kit, the detailed testing procedures of the indicators are as follows: IL-8, TNF-*α*, NT, TXB2, PAF, and VEGF were measured by taking blood from the rat eye. The supernatant was obtained via centrifugation and was tested according to the corresponding kit instructions for standard operation. The AChE was determined as follows: The rat hypothalamus was taken, rinsed with saline, and then blotted with filter paper. 0.3-0.4 g of the sample was homogenized into 10% tissue homogenate, and the homogenate was centrifuged. Then, the supernatant was used to measure AChE. PGE2 and bFGF determination was performed as follows: The rat gastric tissue was taken, rinsed with saline, and then blotted with filter paper. 0.3-0.4 g of the sample was homogenized into 10% tissue homogenate, and the homogenate was centrifuged. Then, the supernatant was used to measure PGE2 and bFGF.

### 2.8. Histopathological Analysis

The rat gastric tissue in each group was collected and opened along the greater curvature. After squeezing out the gastric content, the ulcerated stomachs (3 × 3mm) were washed twice in ice-cold saline, fixed in 10% (V/V) neutral buffered formalin for 5 days, embedded in paraffin wax, sliced into 5 *μ*m thicknesses, deparaffinized in xylene, rehydrated in a decreasing concentration gradient of ethanol, and finally stained with Hematoxylin-Eosin (HE) solution for the examination of histopathology. The observation was conducted with an Olympus microscope-BX60 (Japan Olympus Company, Tokyo, Japan).

### 2.9. Statistical Analysis

All data were checked via two-two comparisons using one-way analysis of variance (ANOVA) LSD and S-N-K with a software package (SPSS16.0). Results were indicated using ($\bar{x}$±s), where s presented the standard deviation.* P* < 0.05 indicates a significant difference, whereas* P* < 0.01 shows an extremely significant difference.

## 3. Results

In order to investigate the effects of Jinlingzi Power decoction and its extracted components on various indexes of the rat's gastric ulcer, blank group (BG), model group (MG), omeprazole group (OG), Jinlingzi powder group extracted using water (JG) were compared with the crude polysaccharide group (C1G), nonalkaloid group (C2G), and alkaloid group (C3G). A serials of rat indexes were measured including UI, UA, UHR, interleukin-8 (IL-8), tumor necrosis factor-*α* (TNF-*α*), neurotensin (NT), platelet activating factor (PAF), thromboxane B2 (TXB2), and vascular endothelial growth factor (VEGF) in rat serum, acetylcholinesterase (AChE) in brain tissue, Prostaglandin E2 (PGE2), and basic fibroblast growth factor (bFGF) in gastric tissue.

### 3.1. Effect on the Index, Area, and Cure Percentage of Rat Ulcer

**Figure 1:**
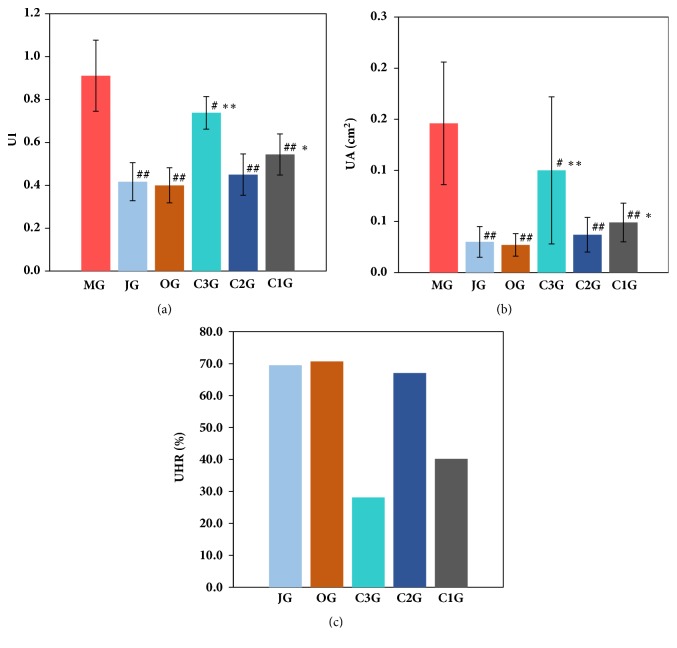
Effect of different groups on UI (a), UA (b), and UHR (c) (results are expressed as the means ± SD. ^#^*P*<0.05* vs* MG, ^##^*P*<0.01* vs* MG, *∗P*<0.05* vs* JG, *∗∗P*<0.01* vs* JG. UI: ulcer index, UA: ulcer area, UHR: ulcer healing rate).

The effects of the Jinlingzi powder decoction and its extracted components on the various indexes are shown in Figure 1 and Table S1 in the Supplementary Materials. As listed in Table S1 and Figure 1(a), the acetic acid induction method successfully formed the gastric ulcer model. After treatment, all groups were more statistically significant than the model group (*P* < 0.05,* P* < 0.01). Among these components, the JG exhibited the best therapeutic effect on the gastric ulcer model (*P* >0.05), and the ulcer index and the area of the ulcer were significantly reduced. It is noticed that therapeutic effect of C2G is similar to that of JG. The gastric ulcer model was cured in the following order: JG = OG > C2G > C1G > C3G. Meanwhile, as displayed in Figure 1(c), the UHR was 67.1% over the C2G, which was almost as effective as the western drug omeprazole (70.7%). Compared with the C1G and C3G, the results were statistically significant (*P* < 0.05,* P *< 0.01), which indicated that the C2G had a significant effect on the treatment of the model ulcer.

**Figure 2:**
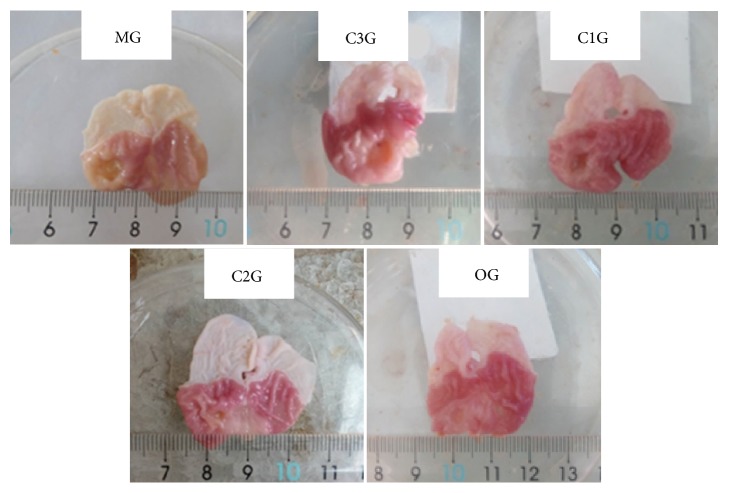
Gastric mucosal injury in rats with acetic acid smear ulcer after treatment by the components of Jinlingzi powder.

Figure 2 shows the gastric mucosal injury in rats with acetic acid smear ulcer after treatment with the components of Jinlingzi powder. In the MG, the mucosa of the gastric antral anterior wall has a single round or circular ulcer surface, a middle depression, a bulge, and a pale central and surrounding gastric mucosa. Additionally, a yellowish white inflammatory necrotic exudate can be found on the surface of the anterior wall of the gastric antrum. Moreover, a flat bottom and a clear boundary were observed, and the mucous membrane around the ulcer was congested and had a more serious edema. After treatment by C1G, C2G, and C3G, the severe gastric mucosal damage caused by acetic acid attenuated effectively. The hyperemia and edema for C3G were still serious, but lighter than those of MG, and the surface had white coating. For C1G, the different degrees of ulcer surface can also be observed, but the ulcer was flatter and the ulcer healed to different degrees. Moreover, the area of the ulcer and its mucosal defect in C1G were smaller than those in the MG. Furthermore, the color of the ulcer was slightly white than that of the surrounding normal tissue. The results of C1G were similar to those of the OG. It was noteworthy that the mucosa in C2G was intact, and the ulcer area evidently became smaller. These results demonstrate that the components of Jinlingzi powers have evident therapeutic actions on acetic acid induced-gastric ulcer; meanwhile, the nonalkaloid component exhibits a remarkable effect.

### 3.2. Effect on the Composition Contents in Rat Serum

**Figure 3:**
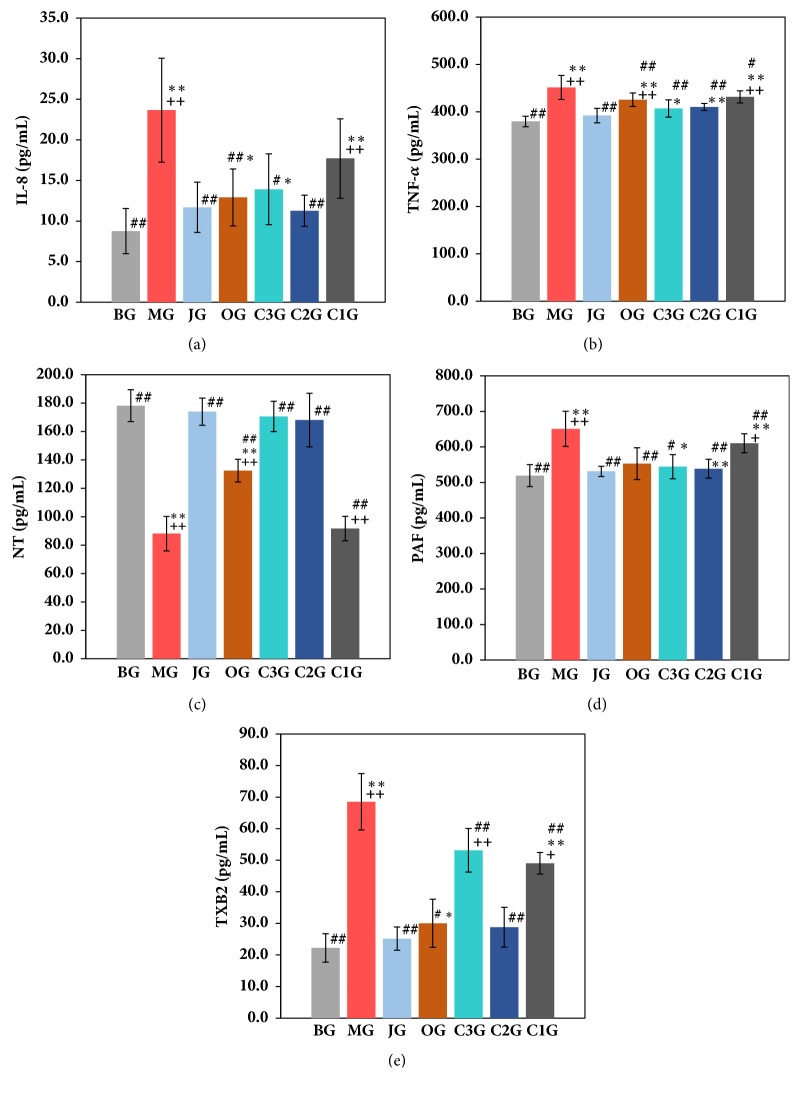
Contents of IL-8 (a), TNF-*α* (b), NT (c), PAF (d), and TXB2 (e) in rat serum ($\bar{x}$±s) (the results are expressed as the means ± SD. ^#^*P*<0.05* vs* MG, ^##^*P*<0.01* vs* MG, *∗P*<0.05* vs* BG, *∗∗ P*<0.01* vs* BG, ^+^*P*<0.05* vs* JG, ^++^*P*<0.01* vs* JG. IL-8: interleukin-8, TNF-*α*: tumor necrosis factor-*α*, NT: neurotensin, PAF: platelet activating factor, TXB2: thromboxane B2).

The effects of the Jinlingzi powder decoction and its extracted components on IL-8, TNF-*α*, NT, PAF, and TXB2 in serum are displayed in Figure 3 and Table S2 in the Supplementary Materials. As listed in Table S2 and Figures 3(a) and 3(b), the amount of IL-8 (Figure 3(a)) and TNF-*α* (Figure 3(b)) evidently increased in the serum of the MG, which is statistically significant (*P* < 0.01) compared with the other groups. The contents of IL-8 and TNF-*α* in the serum of MG were approximately 1.7 times and 0.2 times higher than those of BG, respectively. Notably, the IL-8 content in C1, C2, and C3 groups was 17.71, 11.26, and 13.91 pg/mL, respectively, which is apparently lower than that in the MG after treatment using these components. Similarly, compared with the MG, the TNF-*α* content was also reduced in the C1, C2, and C3 groups although it was not evident. Among these three components, the C2 component shows a major role in decreasing the amounts of IL-8 and TNF-*α*. The above results were in agreement with the effects of JG on the amounts of IL-8 and TNF-*α*. The effects of the C1 component on the decrease in the amounts of IL-8 and TNF-*α* were less than those of the other components (*P* <0.05).

Figure 3(c) and Table S2 display the NT values for each group. Compared with the BG, the NT content of the MG was reduced from 178.19 PGS/mL (BG) to 88.10 pg/mL (MG), which is half the value of the BG (Table S2). The values of NT were 173.99, 170.66, and 168.13 pg/mL for the JG, C2, and C3 groups, respectively, which were similar to that of the BG and far better than that of OG (132.49 pg/mL). The NT contents of the JG, C2, and C3 groups were statistically significant (*P* < 0.01) compared with the MG (Table S2).

As depicted in Table S2 and Figure 3(d), after the acetic acid was applied to the model rats, the PAF content of the MG increased by 25% compared with the BG, indicating that the effect on PAF was not evident. The comparison with the other treatment groups was statistically significant (*P* < 0.05,* P* < 0.01) (Table S2). Meanwhile, the PAF amount of the C2G and C3G decreased by 9.4% and 17%, respectively (*P* < 0.01), which was superior to that of the C1G (6.2%). Results demonstrate that the components of the Jinlingzi powder show relatively small influence on the PAF amount.

Table S2 and Figure 3(e) show the variation of TXB2 data in each experimental group. In the BG group, the TXB2 content was 22.23 pg/mL. The TXB2 amount of the MG increased significantly to 68.52 pg/mL, which was three times higher than that of the BG. The content of TXB2 was reduced by different levels for the JG, OG, C1, C2, and C3 groups. Moreover, the TXB2 content of JG decreased to 25.15 pg/mL, which was close to the value of the BG. For the C1, C2, and C3 groups, the TXB2 amount was 49.02, 28.77, and 53.17 pg/mL, respectively. The results indicate that the nonalkaloids group (C2G) exhibits a more remarkable effect on TXB2, which is statistically significant (*P* < 0.05,* P* < 0.01) compared with the C1G and C3G.

### 3.3. Effect on the AChE Amount in Rat Brain Tissue

Table 1 lists the effects of the various components of the Jinlingzi powder on the AChE content of the rat brain tissue. As shown in Table 1, the AChE content in the brain tissue was reduced from 7.59 IU/mL of BG to 5.38 IU/mL of the MG, which is in accord with the statistical significance (*P* < 0.05,* P* < 0.01) among the groups. The level of AChE was significantly higher in the treatment groups than that of the MG; meanwhile, the order of the AChE content is as follows: JG (7.30 IU/mL) > C3G (7.25 IU/mL) > C2G (6.76 IU/mL) > OG (6.39 IU/mL) > C1G (6.10 IU/mL). It is worth noting that JG showed the best ability to increase the AChE level, even more than the OG value. Additionally, the difference was significant (*P *< 0.05,* P *< 0.01) among the alkaloids, the nonalkaloids, and crude polysaccharide groups. Moreover, alkaloids displayed better AChE content than omeprazole (6.39 IU/mL).

### 3.4. Effect on the Amounts of PGE2 in Rat Gastric Tissue

The effect of Jinlingzi powder decoction and its various components on the PGE2 content in stomach tissue is shown in Table 1. For the MG, the PGE2 content decreased from 59.16 pg/mL to 31.56 pg/mL compared with the BG. The PGE2 contents of the JG, OG, C1G, C2G, and C3G were 57.19, 48.87, 38.84, 55.94, and 40.47pg/mL, respectively. The results suggest that the Jinlingzi powder decoction and nonalkaloids showed a significant effect on the PGE2 amount. Furthermore, the increasing effects of the C3G and C1G on the PGE2 content were not evident and had statistical significance compared with the MG (*P *> 0.05). However, the above results displayed statistical significance (*P *< 0.05) compared with the JG and C3G.

### 3.5. Effect on VEGF Content and VEGFmRNA Expression in Serum

The VEGF content of the MG in rat's serum increased to 3.84 ng/mL from 3.18 ng/mL of the BG (Table 2), but was not statistically significant (*P* > 0.05) compared with the BG. The expression of VEGFmRNA was also enhanced for the MG (Table 2).

Results suggest that the secreted VEGF amount can be increased after acetic acid stimulation. As depicted in Table 2, the VEGF levels were 7.68, 5.83, 5.29, 6.21, and 5.41 ng/mL for the JG, OG, C1G, C2G, and C3G, respectively, and the corresponding expression of VEGFmRNA was also enhanced. The results illustrate that all components had a significant function on the increase in the VEGF content and the expression of VEGFmRNA, which was statistically significant compared with the MG (*P *< 0.05,* P *< 0.01). However, the C2G was not statistically significant compared with the JG (*P* > 0.05).

### 3.6. Effect on bFGF Content and bFGFmRNA Expression in Gastric Tissue

In Table 2, the bFGF content and the bFGFmRNA expression of the rat's stomach tissue increased for the MG, but it was not statistically significant compared with the BG (*P* > 0.05), illustrating that the tissue can increase the secretion of bFGF to some extent after stimulation. Compared with the MG, each treatment group had a significant increase in the bFGF content, suggesting statistical significance (*P* < 0.05,* P* < 0.01). However, the result of C2G was not statistically significant compared with the JG (*P* > 0.05).

### 3.7. Histopathological Examination of Gastric Tissue

**Figure 4:**
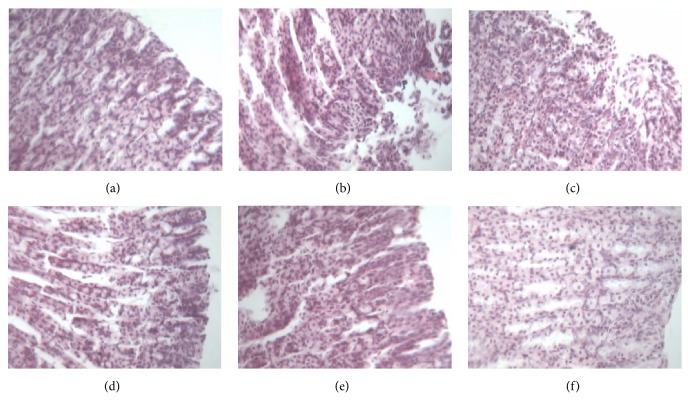
Comparison of the histopathological examination of gastric tissue in each group of rats (HE, × 400 magnification) ((a): BG, (b): MG, (c): C3G, (d): OG, (e): C1G, (f): C2G).

Figure 4 depicts the comparison of the histopathological examination of the gastric tissue of each group of rats. As illustrated in Figure 4(b), the gastric tissue with the acetic acid induced-gastric ulcer (MG) revealed more extensive damage to the gastric mucosa, edema, and leucocyte infiltration compared with those of the BG (Figure 4(a)). For C3G displayed in Figure 4(c), the destroyed antral wall structure and the disordered arrangement of cells could still be found evidently, and many inflammatory cells and exudates could be seen under the microscope. In the OG (Figure 4(d)) and the C1G (Figure 4(e)), it can be observed that the gastric antral mucosa was basically intact, the glands were arranged neatly, and the surface of ulcers was covered with neonate epithelium in varying degrees, suggesting that most ulcers were cured by the C1 component. As shown in Figure 4(f), for the C2G, the antral wall structure was basically recovered; necrosis tissue, mesenchymal hyperemia, and edema basically disappeared; the mucosal gland structure reappeared; the granulation tissue increased in the submucosa; and the mucosa structure was basically restored. Inflammatory cells and exudates disappeared.

Results indicate that the components of Jinlingzi powers can evidently increase mucus production and play a role in the therapeutic actions on the gastric ulcer induced by acetic acid. In summary, among the components of Jinlingzi powder, the C2 showed the best treatment effects on the acetic acid induced-gastric ulcer.

## 4. Discussion

Cytokines IL-8 and TNF-*α* play an important role in acute gastric mucosal injury and have a broad impact on the formation, development, and treatment of ulcer [12, 23–25]. IL-8 is an important chemokine of neutrophil granulocyte, which can induce its morphological change and promote removal of granules, lysokinase release, and protein adhesion [23, 24]. Thus, inflammatory response can be promoted.

TNF-*α* is produced by monocytes and macrophages and is a kind of proinflammatory factor that can cause extensive damage to tissues [12, 25] Generally, a low level of TNF-*α* in plasma plays an important role in maintaining the stability of the internal environment and the organization update, rebuilding the ontogenesis and regulation of immune system. However, excessive amount of TNF-*α* can cause tissue damage. TNF-*α* is also a powerful ulcer-causing medium that can promote the secondary release of other cytokines including IL-1, IL-6, IL-8, PAF, and PGs. Moreover, TNF-*α* can induce neutrophil activation, acute phase protein formation, and small intravascular coagulation during the blood coagulation process. Furthermore, TNF-*α* can influence the supply of mucosal blood oxygen. The above effects will result in the formation of ulcer.

Thus, the values of IL-8 and TNF-*α* in Table S2 and Figures 3(a) and 3(b) show that the gastric ulcer derived from the acetic acid daub type may be formed by inhibiting the coagulation process to start the small intravascular coagulation and by impacting the mucosal blood oxygen supply leading to the increase in the damage in an emergent injury period. Further research is needed to determine which composition plays a key role and how to work in the formation of the gastric ulcer.

NT is a type of brain peptide, and about 85% of NT exists in the stomach and intestine. NT can regulate the various functions of the stomach and intestine. Peptic ulcer is recognized as a result of the stomach and intestine mucosa being digested by the gastric acid and pepsin. Moreover, the functional disturbance of the vagus nerve plays an important role in promoting the formation of ulcer. Therefore, it is helpful to understand the possible mechanism of the drug for the rat ulcer model by measuring the changes of NT in the plasma and the mucosa of the stomach tissue [26].

The NT results in Table S2 and Figure 3(c) indicate that the Jinlingzi powder, nonalkaloid, and alkaloid can exhibit a considerable effect on the increase in the NT content. This result may be related to the effective ingredients having an analgesic effect and an ability to inhibit gastrointestinal motility excitability. In addition, it should also be associated with the effective ingredients being able to inhibit the secretion of gastric acid and pepsin. The study of the experimental animal models showed that the small dose of NT could significantly reduce the production of irritable gastric ulcer in rats [26, 27]. Evers et al. also found that many NT receptors are present in the gastrointestinal tract, suggesting that NT can cause gastrointestinal response, such as the inhibition of gastric acid secretion and gastrointestinal movement, which may be a physiological function [28].

PAF has been regarded as an endogenous phospholipid medium, which exhibits extensive biological activity [29]. PAF can be used as endocrine factors that cause the change of remote organs, and as paracrine and autocrine factors that work for the near tissue and self-tissue cells, respectively. So far, PAF is one of the strongest media that improves the formation of endogenous ulcer. The gastric mucosal damage caused by PAF may be ascribed to the microcirculation disorder and the anti-inflammatory effect that can destroy the mucosa barrier, leading to the relative enhancement of gastric acid erosion. Therefore, measuring the PAF content can help in understanding the microcirculation of the stomach tissue and can further reveal the possible mechanisms of drug therapy for gastric ulcer of model rats [29, 30].

Thromboxane A2 is a derivative of prostaglandin, which has a strong urge for vasoconstriction and platelet aggregation. In general, testing Thromboxane B2 (TXB2) content can reflect the level of the Thrombus A2. The gastric mucosa is able to synthesize and release large amounts of TXA2. When the TXA2 content increases, platelet aggregation can be promoted, causing tiny arterial spasms and capillary thrombosis in the gastric submucosa. Thus, it causes gastric mucosal ischemia, anoxia, and the cell degeneration and necrosis, resulting in the loss of the gastric mucosa barrier. Furthermore, it leads to the diffusion of H^+^ and the formation of gastric ulcer and hemorrhage [31]. The results in Table S2 and Figure 3(e) indicate that the Jinlingzi powder decoction and its nonalkaloid component are beneficial to the reduction of the TXB2 amount, which plays a key role in protecting the gastric mucosa by decreasing the concentration of platelets and the formation of thrombosis. Takahashi et al. also investigated the role of TXA2 in gastric ulcer healing in rats, which confirmed that the effect of TXA2 might be partial due to the prevention of gastric epithelial cell proliferation at the ulcer margin [31].

PAF and TXB2 can cause gastrointestinal mucosa damage, ulcer formation, and stomach bleeding. Moreover, they can also produce inflammatory mediators and have synergistic effects with inflammatory factors such as TNF and IL leading to the vascular tissue damage. The experimental results in Figure 3 and Table S2 demonstrate that Jinlingzi powder decoction and the nonalkaloids can increase gastric mucosal blood flow and relieve gastric submucosa small artery spasm and capillary vessel thrombosis.

The influences of Jinlingzi powder decoction and its various components on the amounts of IL-8, TNF-*α*, NT, PAF, and TXB2 in the serum of the model rats demonstrate that the IL-8 content is the most evident change, followed by TXB2 and then NT, PAF, and TNF-*α*. Among the various groups, the Jinlingzi powder decoction and the nonalkaloid components cause the considerable changes of the above factors in the serum. Moreover, their effects are similar. Therefore, it is concluded that the nonalkaloid components of the Jinlingzi powder can effectively inhibit the neutral neutrophil activation, prevent the capillary thrombosis, and protect the gastric mucosa. Thus, the nonalkaloid components of the Jinlingzi powder play a key role in the treatment of gastric ulcer.

AChE is a kind of hydrolase that mostly exists in the nervous system. AChE not only participates in the delivery of cholinergic neurotransmitters, but also regulates and promotes neural tissue development and nerve regeneration. Peptic ulcer is believed to be caused by the digestion of gastric acid and pepsin on the gastrointestinal mucosa. Moreover, the disturbance of the vagus nerve function plays an important role in promoting ulcer [32]. Therefore, detecting the neurotransmitter content of the neurons in the brain tissue can reveal the possible treatment mechanism of the drug in the gastric ulcer model of the rat. The tested AChE results in Table 1 demonstrate that ulcer healing may be related to the effect of the effective ingredients in pain, suppressing the stimulation of the gastrointestinal tract, and the secretion of the stomach acid and pepsin.

PGE2 is highly expressed in the gastric mucosa and has a variety of important physiological functions. PGE2 can not only inhibit the secretion of gastric acid and stimulate tissue repair process to promote ulcer healing, but also stabilize mast cell membrane and inhibit the release of cytotoxic substances, such as PAF, TNF, and lysosomal enzyme. Therefore, it is beneficial to ulcer healing [33, 34]. As displayed in Table 1, the PGE2 values suggest that ulcer healing over the components of the Jinlingzi powder may be related to its anti-cell protective effect by promoting mucus secretion, enhancing the mucus barrier, and improving mucosal blood flow, which can inhibit gastric acid secretion and stimulate tissue repair by increasing the protection mechanism to promote the healing of ulcer.

To confirm the inhibition of gastric acid secretion, a pyloric ligation ulcer model was also selected as one of the research models. Fig.  S1(left) in the Supplementary Materials displays the UHR on the gastric ulcer of pyloric ligation type over Jinlingzi powder and its components. Jinlingzi powder and its components show the preventive and therapeutic effects of pyloric ligation type on gastric ulcer; meanwhile, the C2G also exhibits a better UHR on the gastric ulcer of pyloric ligation type, although the UHR value is lower than that of OG. Compared with Fig.  S1(right), the curative effect on pyloric ligation model is much worse than that on the acetic acid induced-gastric ulcer. Thus, we suggest that Jinlingzi powder and its components can inhibit acid secretion to a certain extent, but the effect is not prominent. On the basis of the above results, acetic acid induced-gastric ulcer was selected as experimental model of gastric ulcer to investigate in detail the therapeutic actions of Jinlingzi powder and its components.

In addition, as shown in Figure 4, the histopathological examinations of gastric tissue in each group of rats also confirm that the components of Jinlingzi powder can evidently increase mucus production.

VEGF is considered as a multifunctional cytokine for the endothelial cells of the blood vessel [35]. Generally, VEGF has a high concentration in the gastric juice, which can promote the proliferation of the mucosal epithelium. Inside and outside the body, it can stimulate the synthesis of DNA and RNA for multiple cells, maintain the integrity of the gastrointestinal mucosa, and significantly increase the blood flow of the gastric mucosa. Therefore, the influences of VEGF on tissue repair and vascular regeneration have attracted much more attention of researchers [35–37]. Results in Table 2 indicate that the nonalkaloids (C2) exhibit a better effect on the increase in VEGF content than other groups, which may be due to its powerful promotion in the formation of blood vessels and production of granulation tissue, resulting in the rapid healing of gastric ulcer to protect the gastric mucosa.

bFGF is a kind of cationic polypeptide growth factors which is composed of 146 amino acids. bFGF is widely dispersed in various tissues of the human and has a broad range of physiological functions. bFGF is also an important wound-healing factor that can affect the entire wound repair process, which promotes angiogenesis, especially in the formation of granulation tissue. Additionally, it plays a decisive role in the treatment of ulcers and the quality of their healing [7–9]. To sum up, the nonalkaloid group presents better increase in bFGF content than the other groups (Table 2). This performance may be related to the active ingredients contained in the organization, which plays a powerful effect on promoting blood vessels, indirectly enhancing the synthesis and secretion of collagen fibers and other mechanisms in the granulation tissue. Thus, the maturity of granulation tissue is improved, thereby increasing the healing quality. bFGF as a chemokine can directly promote the tissue cells that repair the damaged areas.

Soker et al. found that bFGF and VEGF displayed a powerful role in promoting the formation of angiogenesis in local ischemic animal models [38]. Szaho et al. studied endogenous bFGF expression in the healing process of gastric ulcer. They found that it was weakly positive in the normal gastric mucosa, strongly positive in the acute stage of gastric ulcer, and gradually weak in the healing stage of gastric ulcer [8].

The experimental results of the pharmacological indexes show that the nonalkaloid (C2G) displays a better function on the repair and healing of ulcer, and their treating effect is even better than omeprazole (OG) in some indexes for rats (Figures 1 and 3).

Moreover, the effects of nonalkaloids are similar to that of the Jinlingzi powder decoction for various pharmacological indexes, which suggests that the treatment of gastric ulcer via Jinlingzi powder may be performed by nonalkaloids. In addition, alkaloids may also play an important role in the change of the NT and PAF contents in the serum and the AChE amount in the brain. Moreover, alkaloids could have a better analgesic effect through neural regulation, thereby reducing abdominal pain symptoms derived from gastric ulcer.

## 5. Conclusion

In general, Jinlingzi powder and its nonalkaloids have an overall effect on treating the gastric ulcer model induced by acetic acid, which may be attributed to the following: (1) Reducing the content of IL-8 and TNF-*α* inhibits small intravascular coagulation during the blood coagulation process and the blood oxygen supply of mucosa during the acute injury stage. (2) Enhancing the content of NT and AChE has an analgesic effect, inhibiting gastrointestinal motility and secretion of excitatory transmitters, gastric acid, and pepsin. (3) Decreasing PAF and TXB2 contents can increase the blood flow volume of the gastric mucosa and alleviate the spasm of the arteriole and the formation of capillary thrombosis in the submucosa of the stomach to protect the gastric mucosa. (4) Promoting PGE2 content in the gastric tissue protects the cells by promoting the secretion of mucus, enhancing the mucus barrier, increasing the blood flow of the mucous membrane. and controlling the secretion of gastric acid to stimulate tissue repair and promote ulcer healing. (5) Increasing the amount of VEGF and bFGF and their expression can enhance the formation of blood vessels and granulation tissues to protect the gastric mucosa and to speed up ulcer healing.

In summary, Jinlingzi powder and its nonalkaloid components can speed up gastric ulcer healing. The effect of treating gastric ulcer is closely related to effective constituents. In the future, systematic investigation on the chemical compositions of nonalkaloids is necessary to illuminate its pharmacodynamic function. Moreover, the content and fingerprint of different index components in nonalkaloids should also be measured to establish a reasonable and controllable quality standard, thereby establishing a massy theoretical and experimental foundation for the research of Jinlingzi powder.

## Figures and Tables

**Table 1:** AChE content of the brain tissue and the PGE2 contents of gastric tissue ($\bar{x}$±s).

Group	Number of rats	AChE (IU/mL)	PGE_2_ (pg/mL)
BG	12	7.59 ± 0..41^##^	59.16 ± 3.18^##^
MG	9	5.38 ± 0.24^*∗∗*++^	31.56 ± 2.36^*∗∗*++^
JG	10	7.30 ± 0.47^##^	57.19 ± 3.57^##^
OG	11	6.39 ± 0.46^##*∗*+^	48.87 ± 4.51^##^
C3G	9	7.25 ± 0.67^##^	40.47 ± 4.43^*∗*##+^
C2G	10	6.76 ± 0.35^##^	55.94 ± 2.93^##^
C1G	10	6.10 ± 0.48^#*∗∗*++^	38.84 ± 2.85^*∗∗*+^

The results are expressed as the means ± SD. ^#^*P*<0.05 *vs* MG, ^##^*P*<0.01 *vs* MG, *∗P*<0.05 *vs* BG, *∗∗P*<0.01 *vs* BG, ^+^*P*<0.05 *vs* JG, ^++^*P*<0.01 *vs* JG. AChE: acetylcholinesterase; PGE2: prostaglandin E2.

**Table 2:** VEGF content of serum, bFGF content of gastric tissue, and the expression intensity of VEGFmRNA and bFGFmRNA ($\bar{x}$±s).

Group	Number of rats	VEGF (ng/mL)	bFGF (pg/mL)	VEGFmRNA	bFGFmRNA
BG	12	3.18 ± 0.41	105.82 ± 11.89	0.098 ± 0.011	0.75 ± 0.034
MG	9	3.84 ± 0.44	110.77 ± 13.94	0.212 ± 0.013	0.91 ± 0.101
JG	10	7.68 ± 1.62^##*∗∗*^	142.13 ± 19.53^##*∗∗*^	0.511 ± 0.022^##*∗∗*^	1.62 ± 0.077^##*∗∗*^
OG	11	5.83 ± 0.67^##*∗∗*^	122.99 ± 8.38^##*∗∗*^	0.432 ± 0.019^##*∗∗*^	1.39 ± 0.075^##*∗∗*+^
C3G	9	5.41 ± 0.63^##*∗∗*+^	123.19 ± 9.77^##*∗∗*+^	0.411 ± 0.037^##*∗∗*+^	1.19 ± 0.067^##*∗∗*++^
C2G	10	6.21 ± 0.77^##*∗∗*^	131.93 ± 12.64^##*∗∗*^	0.455 ± 0.035^##*∗∗*^	1.46 ± 0.035^##*∗∗*^
C1G	10	5.29 ± 0.51^##*∗∗*++^	119.41 ± 17.44^##*∗∗*++^	0.398 ± 0.031^##*∗∗*++^	1.24 ± 0.048^##*∗∗*++^

The results are expressed as the means ± SD. ^#^*P*<0.05 *vs* MG, ^##^*P*<0.01 *vs* MG, *∗P*<0.05 *vs* BG, *∗∗P*<0.01 *vs* BG, ^+^*P*<0.05 *vs* JG, ^++^*P*<0.01 *vs* JG. VEGF: vascular endothelial growth factor; bFGF: basic fibroblast growth factor.

## Data Availability

The data used to support the findings of this study are included within the article. More detailed description and explanation are available from the corresponding author upon request.

## Supplementary Materials

Supplementary Materials Table S1 lists the data of UI, UA, and UHR over different treatment groups; Table S2 presents the contents of IL-8, TNF-*α*, NT, PAF, and TXB2 in rat serum over different treatment groups; and Fig. S1 shows UHR on the gastric ulcer of pyloric ligation type (left) and acetic acid smear type (right) over Jinlingzi powder and its components.
