# Supplementary material for: Treatment Effects of Jinlingzi Powder and Its Extractive Components on Gastric Ulcer Induced by Acetic Acid in Rats
Source: Evid Based Complement Alternat Med. 2019 Jan 3;2019:7365841. doi: 10.1155/2019/7365841 (PMC6335817; doi:10.1155/2019/7365841)
Supplement: Supplementary Materials — Table S1 lists the data of UI, UA, and UHR over different treatment groups; Table S2 presents the contents of IL-8, TNF-α, NT, PAF, and TXB2 in rat serum over different treatment groups; and Fig. S1 shows UHR on the gastric ulcer of pyloric ligation type (left) and acetic acid smear type (right) over Jinlingzi powder and its components. [file 7365841.f1.pdf]

Supplementary Information

Treatment Effects of Jinlingzi Powder and its Extractive Components on Gastric Ulcer Induced by Acetic Acid in Rats

Xueying Zhao*, Ji Li, Yonghai Meng, Mingming Cao, Jianwei Wang*

School of Basic Medical Sciences, Heilongjiang University of Chinese Medicine, 24 Heping Road, Harbin, 150040, China

Table S1 effect of different groups on UI, UA and UHR

Group	Number of rats	UI	UA (cm^2)	UHR (%)
BG	12	0	0	—
MG	9	0.911±0.166	0.146±0.060	—
JG	10	0.417±0.089[##]	0.030±0.015[b]	69.5
OG	11	0.400±0.082[##]	0.027±0.011[b]	70.7
C3G	9	0.738±0.076[#**]	0.100±0.072[ad]	28.1
C2G	10	0.450±0.096[##]	0.037±0.017[b]	67.1
C1G	10	0.544±0.096[##*]	0.049±0.019[bc]	40.2

The results are expressed as the means ± SD. [#] *P*<0.05 *vs* MG, [##] *P*<0.01 *vs* MG, * *P*<0.05 *vs* JG, ** *P*<0.01 *vs* JG. (UI: ulcer index, UA: ulcer area, UHR: ulcer healing rate)

Table S2 contents of IL-8, TNF-α, NT, PAF and TXB2 in rat serum ($\bar{x}\pm s$)

Group	Number of rats	IL-8 (pg/mL)	TNF-α (pg/mL)	NT (pg/mL)	PAF (pg/mL)	TXB2 (pg/mL)
BG	12	$8.75\pm2.78^{\#\#}$	$379.65\pm11.11^{\#\#}$	$178.19\pm11.24^{\#\#}$	$519.29\pm30.97^{\#\#}$	$22.23\pm4.48^{\#\#}$
MG	9	$23.66\pm6.404^{**++}$	$451.36\pm25.47^{**++}$	$88.10\pm12.20^{**++}$	$651.18\pm49.39^{**++}$	$68.52\pm8.93^{**++}$
JG	10	$11.69\pm3.09^{\#\#}$	$391.95\pm15.33^{\#\#}$	$173.99\pm9.58^{\#\#}$	$531.41\pm14.33^{\#\#}$	$25.15\pm3.69^{\#\#}$
OG	11	$12.91\pm3.51^{\#\#*}$	$425.43\pm14.39^{\#\#**++}$	$132.49\pm7.97^{\#\#**++}$	$553.07\pm44.72^{\#\#}$	$30.00\pm7.64^{\#*}$
C3G	9	$13.91\pm4.36^{\#*}$	$407.06\pm18.26^{\#\#*}$	$170.66\pm10.71^{\#\#}$	$544.46\pm34.02^{\#}$	$53.17\pm6.92^{**++}$
C2G	10	$11.26\pm1.92^{\#\#}$	$410.06\pm7.25^{\#\#**}$	$168.13\pm18.90^{\#\#}$	$538.62\pm26.56^{\#}$	$28.77\pm6.30^{\#\#}$
C1G	10	$17.71\pm4.89^{**++}$	$431.43\pm12.95^{\#**++}$	$91.72\pm8.68^{**++}$	$610.62\pm26.88^{\#**++}$	$49.02\pm3.41^{\#\#**+}$

The results are expressed as the means ± SD. $^{\#}$ *P*<0.05 *vs* MG, $^{\#\#}$ *P*<0.01 *vs* MG, * *P*<0.05 *vs* BG, ** *P*<0.01 *vs* BG, $^{+}$ *P*<0.05 *vs* JG, $^{++}$ *P*<0.01 *vs* JG. IL-8: interleukin-8, TNF-α: tumor necrosis factor-α, NT: neurotensin, PAF: platelet activating factor, TXB2: thromboxane B2.

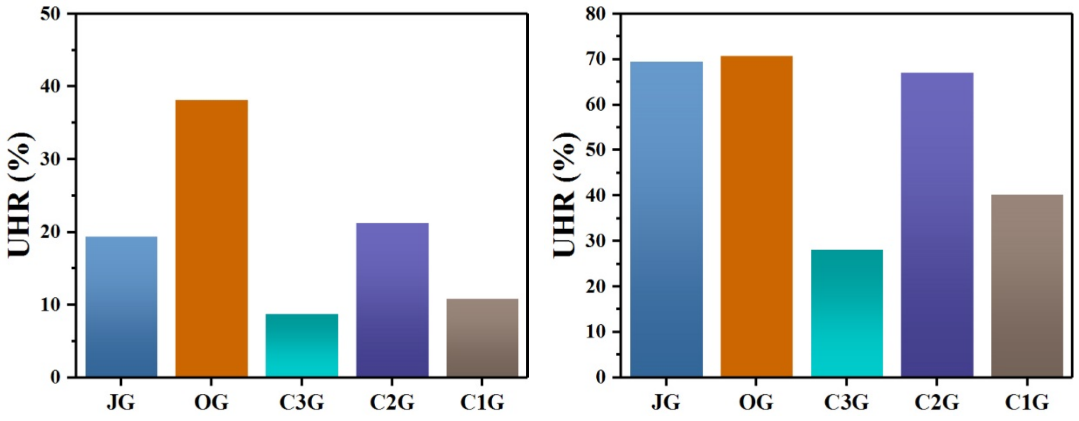

Fig. S1 UHR on the gastric ulcer of pyloric ligation type (left) and acetic acid smear type (right) over Jinlingzi Powder and its components (Ten rats in each group)
